# GLYNAC SUPPLEMENTATION LOWERS POSTPRANDIAL INFLAMMATION AND OXIDATIVE STRESS TO PROMOTE HEALTH IN OLDER HUMANS

**DOI:** 10.1093/geroni/igae098.0415

**Published:** 2024-12-31

**Authors:** Rajagopal Sekhar, Premranjan Kumar, George Taffet, Charles Minard

**Affiliations:** Baylor College of Medicine, Houston, Texas, United States; Baylor College of Medicine, Houston, Texas, United States; Houston Methodist Hospital, Houston, Texas, United States; Baylor College of Medicine, Houston, Texas, United States

## Abstract

Elevated oxidative stress (OS) and inflammation are linked to several age-related abnormalities, but factors contributing to these defects are not well understood. Meal consumption is a known stimulus of OS and inflammation. In a randomized clinical trial (RCT) we investigated the impact of a single carbohydrate test-meal on postprandial OS and inflammation in older adults (OA) and young adults (YA), and tested the effect of supplementing GlyNAC (glycine and N-acetylcysteine) vs. placebo. 20 OA and 10 YA received a 75g oral glucose-test-meal with assessment of 2-hour postprandial change in plasma markers of OS (as TBARS) and inflammation (as IL-6). These measures were repeated and compared after OA and YA received their assigned supplements. Pre-supplementation: After consuming the glucose-meal, OA had significantly higher 2-hour postprandial increases in OS (change in plasma concentrations of TBARS: YA 0.02 ± 0.19 vs. 1.09 ± 0.96 in OA, p=0.03) and inflammation (change in plasma concentrations of IL-6: YA 0.03 ± 0.03 vs. 0.52 ± 0.22 OA, p<0.001). Post-supplementation: GlyNAC protected OA from the glucose-meal driven increases in OS (TBARS pre: 1.03 ± 0.44 vs. post -0.04 ± 0.19, p<0.001) and inflammation (IL-6 pre: 0.52 ± 0.20 vs. post 0.05 ± 0.03, p<0.001). No effects occurred in OA receiving placebo or YA receiving GlyNAC. These data indicate that carbohydrate intake contributes to significant increases in postprandial OS and inflammation in OA. By protecting from the postprandial (and overall) rise in OS and inflammation after carbohydrate consumption, GlyNAC supplementation promotes postprandial health in older humans.

